# Anterior temporal lobe is necessary for efficient lateralised processing of spoken word identity

**DOI:** 10.1016/j.cortex.2019.12.025

**Published:** 2020-01-24

**Authors:** Thomas E. Cope, Yury Shtyrov, Lucy J. MacGregor, Rachel Holland, Friedemann Pulvermüller, James B. Rowe, Karalyn Patterson

**Affiliations:** a Department of Clinical Neurosciences, University of Cambridge, UK; b MRC Cognition and Brain Sciences Unit, University of Cambridge, UK; c Center of Functionally Integrative Neuroscience, Aarhus University, Denmark; d Institute for Cognitive Neuroscience, NRU Higher School of Economics, Moscow, Russia; e Division of Language and Communication Science, City University London, UK; f Brain Language Laboratory, Department of Philosophy and Humanities, WE4, Freie Universität Berlin, Germany

**Keywords:** Language, Semantic, Anterior temporal lobe, Magnetoencephalography, Laterality

## Abstract

In the healthy human brain, the processing of language is strongly lateralised, usually to the left hemisphere, while the processing of complex non-linguistic sounds recruits brain regions bilaterally. Here we asked whether the anterior temporal lobes, strongly implicated in semantic processing, are critical to this special treatment of spoken words. Nine patients with semantic dementia (SD) and fourteen age-matched controls underwent magnetoencephalography and structural MRI. Voxel based morphometry demonstrated the stereotypical pattern of SD: severe grey matter loss restricted to the anterior temporal lobes, with the left side more affected. During magnetoencephalography, participants listened to word sets in which identity and meaning were ambiguous until word completion, for example PLAYED versus PLATE. Whereas left-hemispheric responses were similar across groups, patients demonstrated increased right hemisphere activity 174–294 msec after stimulus disambiguation. Source reconstructions confirmed recruitment of right-sided analogues of language regions in SD: atrophy of anterior temporal lobes was associated with increased activity in right temporal pole, middle temporal gyrus, inferior frontal gyrus and supramarginal gyrus. Overall, the results indicate that anterior temporal lobes are necessary for normal and efficient lateralised processing of word identity by the language network.

## 1 Introduction

The neural processing of spoken words is strongly lateralised to the dominant cerebral hemisphere, usually the left, while the processing of complex non-linguistic sounds recruits brain regions bilaterally (Shtyrov, Kujala, Palva, Ilmoniemi, & Näätänen, 2000; Tervaniemi & Hugdahl, 2003; Zatorre et al., 1992, 2002). Across a range of primate species, acoustic information entering primary auditory cortex is rapidly transferred along reciprocal connections to the anterior temporal lobe (ATL) (Friederici, 2012; Hackett, 2011), a region that is strongly implicated in the representation and processing of semantic information in the human brain (Binder et al., 2011; Binney, Embleton, Jefferies, Parker, & Lambon Ralph, 2010; Guo et al., 2013; Lambon Ralph, Jefferies, Patterson, & Rogers, 2017; Mion et al., 2010; Mummery et al., 2000; Pobric, Jefferies, & Lambon Ralph, 2007; Visser, Jefferies, & Lambon Ralph, 2010). The disambiguation of word-endings recruits language-specific brain regions, and has previously been demonstrated to produce a strongly left-lateralised response in young, healthy listeners (Holland, Brindley, Shtyrov, Pulvermu¨ ller, & Patterson, 2012). This left-lateralisation is most prominent between 150 and 350 milliseconds after the stimulus, the time windows that are generally considered to reflect the early automatic analysis of linguistic information (MacGregor, Pulvermu¨ ller, Van Casteren, & Shtyrov, 2012). Later cognitive processing of the meaning of language, first reflected in the N400 (300–500 msec) response, is typically symmetric over the hemispheres or even right lateralized (Kutas & Federmeier, 2011).

Here we recorded neural activity with magnetoencephalography (MEG) while participants listened to word sets in which identity and meaning were ambiguous until word completion, for example PLAYED versus PLATE. We compared neural responses between healthy participants and people with neurodegeneration of ATL due to semantic dementia (SD, also known as the semantic variant of primary progressive aphasia, a type of frontotemporal dementia). The advantage of MEG in this context is that it allowed us to compare the time-course of neural activity between these two groups with sufficient spatial resolution to assess the approximate location of simultaneously-active brain regions. MEG has been shown to be sensitive to both semantic decisions (Hughes, Nestor, Hodges, & Rowe, 2011) and auditory change detection abnormalities (Hughes, Ghosh, & Rowe, 2013; Hughes & Rowe, 2013) in frontotemporal dementia. We employed a spoken-word version of the auditory mismatch paradigm (for a review see Näätänen, Paavilainen, Rinne, & Alho, 2007), in which repeated ‘standard’ words (for example PLAY) were changed in either grammatical category (tense) or semantic meaning by the spliced addition of the additional endings d/t (to become, in this case, PLAYED or PLATE). This paradigm is a sensitive tool for measuring automatic lexico-semantic processing of spoken words in the brain (Pulvermüller, Shtyrov, Ilmoniemi, & Marslen-Wilson, 2006; Shtyrov, Kujala, & Pulvermüller, 2010) and has a special benefit for patient studies as it does not require any active stimulus processing, or even attention on the auditory stream (Gansonre, Højlund, Leminen, Bailey, & Shtyrov, 2018). Here, presentation was designed such that the occurrence and timing of a deviant word were predictable, but the identity and meaning of the word were unpredictable until the last tens of milliseconds of its utterance. This allowed us to examine specifically the processing, not just of words in general, but of those aspects of word processing that are to do with semantic identity and meaning.

We asked whether the integrity of anterior temporal lobes is necessary for the lateralised processing of spoken word identity in extra-temporal brain regions. This central question was motivated in part by a clinical observation. Neurodegeneration of the anterior temporal lobes, generally more severe in the dominant (usually left) hemisphere, results in the clinical syndrome of semantic dementia (SD). SD erodes semantic memory and conceptual knowledge as well as language function (Bozeat, Lambon Ralph, Patterson, Garrard, & Hodges, 2000; Patterson et al., 2006; Warrington, 1975), in keeping with emerging views of ATL as a transmodal semantic hub (Guo et al., 2013; Lambon Ralph et al., 2017; Patterson, Nestor,& Rogers, 2007). In SD, processing of single spoken words at the acoustic/phonetic level is entirely adequate to enable repetition: if you ask an SD patient to repeat a long and complicated word like “hippopotamus”, they will typically do so correctly and effortlessly. But, ask the patient what a hippopotamus is, and the response from a mild case might be: “is it some sort of animal?” and from a moderate or severe case: “I don't know”. Importantly, patients with SD may also struggle to repeat longer sequences of words or sentences, frequently displaying phonemic exchanges (e.g., “The *flag blew* in the wind” repeated as “The *blag flew* in the wind”), especially if the word sequence/sentence contains infrequently encountered words (Patterson, Graham, & Hodges, 1994; Warrington & McCarthy, 1987). Similarly, despite relatively preserved day-to-day episodic and prospective memory, patients with SD sometimes struggle on tests of delayed recall, producing answers that ‘sound-like’ the information they were asked to retain. A recent patient, asked to retain the name-and-address from the Addenbrooke's Cognitive Examination: “Harry Barnes, 73 Orchard Close, Kingsbridge, Devon”, recalled ten minutes later: “Harry Buns, 73 Awkward Close, I've forgotten the rest.”

These response patterns suggest that, with degeneration of the anterior temporal lobe, patients might be encoding information phonetically rather than lexically (Papagno, Vernice, & Cecchetto, 2013) (for a review of this distinction see Snowling, Chiat, & Hulme, 1991; Gathercole, 1995). This leads to poorer recall performance for words that are no longer understood (Knott, Patterson, & Hodges, 1997; Patterson et al., 1994), as patients lose the normal recall benefit for real words over non-words that is observed in healthy participants (Hulme, Maughan, & Brown, 1991). Indeed, there is evidence that in SD, the brain processing of real words and word-like non-words becomes increasingly similar. For example, SD patients are impaired at distinguishing between real words and non-words in a visual lexical decision task, especially if the non-word in a word/non-word pair (such as FRUIT/FRUTE) follows a more typical orthographic pattern than the word, as measured by bigram and trigram frequencies (Patterson et al., 2006; Rogers, Lambon Ralph, Hodges, & Patterson, 2004). Similarly, patients with SD are relatively impaired at identifying acoustically degraded speech in categories for which they have impaired semantic knowledge (place names), compared to those for which their knowledge is intact (number strings) (Hardy et al., 2018), and indeed generally show a striking advantage in verbal working memory for numbers compared to other word-types (Jefferies, Patterson, Jones, Bateman, & Lambon Ralph, 2004).

SD is characterised by progressive deterioration of conceptual knowledge, modulated by familiarity. Because it is a central semantic disorder, the cognitive impact is not confined to language; but language deficits are early and prominent, leading to an additional characterisation of the condition within the spectrum of primary progressive aphasias as the semantic variant (Gorno-Tempini et al., 2011). Deficits in confrontational naming and word comprehension are especially prominent, whereas repetition, grammar, and motor speech are usually well preserved until late in the illness. The syndrome results from neurodegeneration of anterior temporal lobes that is usually more severe in the left hemisphere, and is almost always caused by TDP-43 type-C neuropathology (Hodges et al., 2009; Rohrer et al., 2011; Spinelli et al., 2017). By the time of clinical presentation, this temporal lobe neurodegeneration is usually already severe, and even patients at a moderate stage of illness and living relatively normal daily lives may show 50e80% loss of left anterior temporal grey matter (Hodges & Patterson, 2007). However, this atrophy is also confined to the temporal lobes, meaning that any changes in the neural responses observed in extra-temporal language regions represent diaschisis in intact cortex. Longitudinal imaging studies, employing boundary shift integrals (Rohrer et al., 2008) and tensor based morphometry (Brambati et al., 2009), have demonstrated grey matter atrophy in SD (compared to controls) only in the temporal lobes. This was confirmed by Bocchetta et al. (2019), using a subregion segmentation to demonstrate the atrophy in SD spreading from the temporal poles, involving other cortical regions only in very late disease.

The fact that SD patients can perform an ‘off-line’ task like listening to and repeating a spoken word does not establish that the earliest stages of spoken-word processing in SD are unaltered. In the healthy brain, early processing, whilst not unilateral, is biased towards the left hemisphere with increasing left-lateralisation observed as information moves forward from posterior to anterior regions (Marinkovic et al., 2003). Here we directly tested how the pattern of neural activity involved in processing the identity of spoken words is affected by disruption of the reciprocal connectivity between undamaged early auditory regions in posterior superior temporal lobe and severely compromised transmodal semantic regions in ATL. Specifically, our analyses of the MEG data from SD patients relative to healthy age-matched controls addressed the question of whether degeneration of the ATL would result in disruption of the normal pattern of laterality in spoken identity word processing. We hypothesised that we would observe a shift from a left-dominant pattern in controls to bilateral activation of the language network, as more widespread acoustic processing is engaged to compensate for the loss of normal, efficient, semantic mechanisms. Specifically, our hypothesis predicts diaschisis: the consequence of anterior temporal lobe atrophy is seen as a change in activity elsewhere in the brain.

## 2 Methods

### 2.1 Participants

Eleven patients with semantic dementia (SD) were recruited from a single tertiary referral cognitive clinic. All patients met consensus diagnostic criteria for both SD (Neary et al., 1998) and semantic variant primary progressive aphasia (Gorno-Tempini et al., 2011). Nine of the patients (eight right-handed, one left-handed) tolerated the MEG environment sufficiently to complete the whole experimental paradigm, and provided the data reported here. Eight were able to undertake a research structural MRI brain scan. The sample size was limited by the availability of patients with this rare disease who were able to give informed consent to both MRI and MEG scanning, with the explicit acknowledgement that this number would allow sufficient power to detect only large effects.

Fourteen right-handed, healthy individuals of a similar age were recruited as controls. All produced complete MEG datasets and underwent a structural MRI head scan.

Participant demographics are shown in Table 1. Single subject atrophy patterns are shown in supplementary figure 1, and case vignettes for each subject are provided in supplementary materials.

Study procedures were approved by the UK National Research Ethics Service. All participants had mental capacity and gave written informed consent to participation in the study.

### 2.2 Experimental paradigm

The procedure closely mirrored that of a previously published MEG study of the hemispheric laterality of word processing in healthy young adults (Holland et al., 2012). Participants sat upright in a magnetically shielded room, watching a silent movie while passively listening to spoken words delivered through an in-ear air tube system. Before the commencement of MEG recording, a single-frequency (1 kHz) pure-tone audiogram was performed through the air tube sound delivery system to ensure that stimuli were audible at a comfortable level in both ears and not impeded by kinks in the tubing, or by participant hearing impairment. During the primary experiment, no response was required, thereby reducing the difficulties inherent in the comprehension and retention of a behavioural task for patients with semantic impairment.

**Fig. 1:**
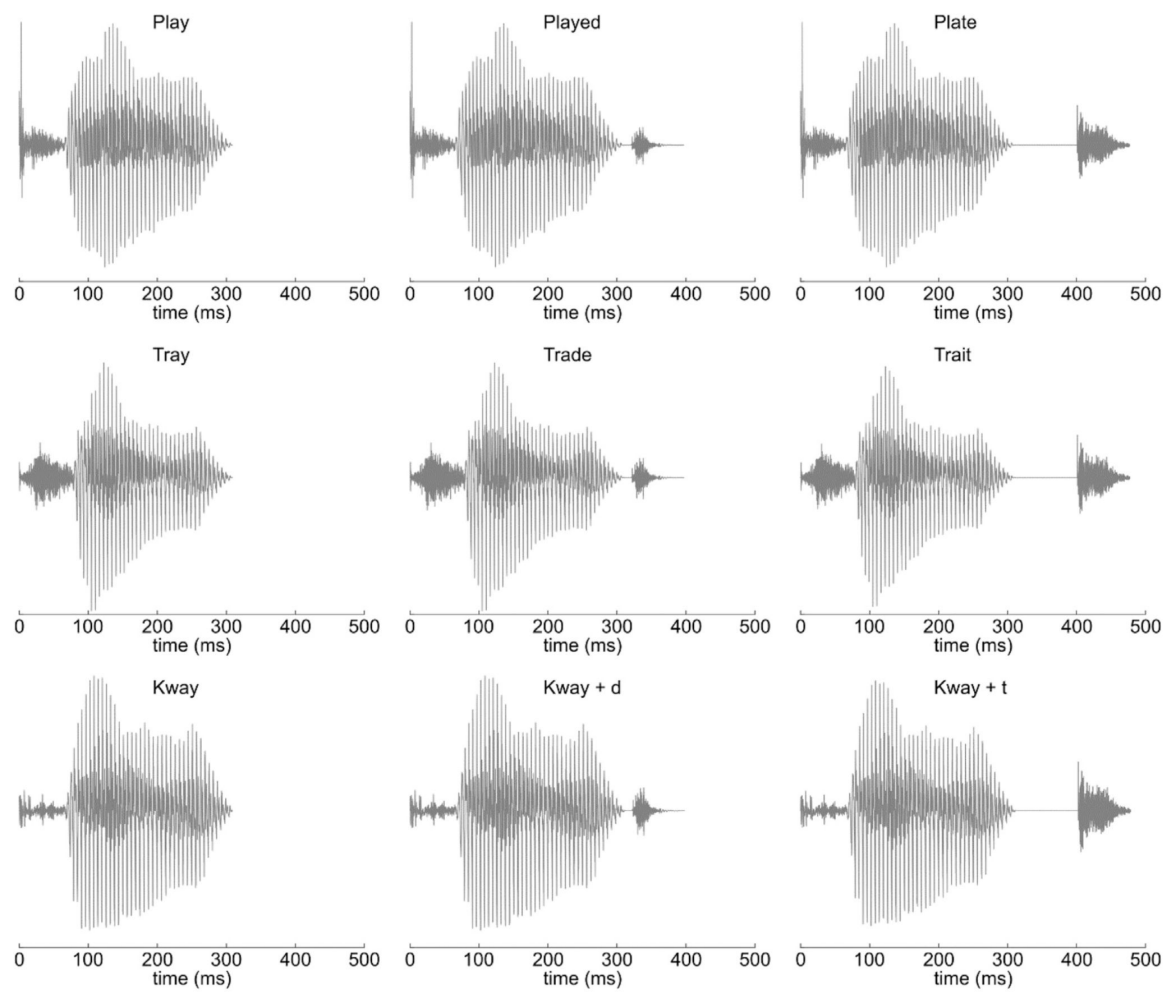
Waveforms of the three standard words, with the spliced addition of/d/and/t/deviant endings. All stimuli within each triplet were identical for the first 320 msec.

Words consisted of one of three standard (template) words and two deviants for each standard that varied in their endings (Fig. 1). Standards comprised the real words ‘PLAY’ and ‘TRAY’, and the pseudo-word ‘KWAY’, all closely matched acoustically and phonetically. Deviant endings were created by the spliced addition of/d/or/t/to the end of a standard word, avoiding coarticulation effects and resulting in the six deviant stimuli ‘PLAYED’, ‘PLATE’; ‘TRADE’, ‘TRAIT’; and ‘KWAYED’ (or ‘KWADE’), ‘KWATE’. This acoustic splicing avoided coarticulation effects without sounding unnatural, and resulted in a divergence point between/d/and/t/endings 10 msec after the offset of the standard word. Audio files of the stimuli are available as supplementary materials to this article.

Presentation followed a repeating pattern of 4 standards to 1 randomly chosen deviant, with a fixed 1 sec inter-onset-interval, such that the *occurrence* of a deviant was entirely predictable but its *identity* was not. For example, after four presentations of the word ‘PLAY’, the next word would be either ‘PLAYED’ or ‘PLATE’. Stimuli were presented in blocks such that each participant heard a single template word 800 times and each of its deviant forms 100 times. Blocks therefore lasted 1000 sec (approximately 17 min), and the order of presentation was counterbalanced across participants.

### 2.3 Voxel based morphometry

Eight patients with SD and 14 controls underwent structural MR imaging using a 3 T Siemens Magnetom Tim Trio scanner with a 32-channel phased-array head coil. A T1-weighted magnetisation-prepared rapid gradient-echo (MPRAGE) image was acquired with repetition time (TR) = 2250–2300 msec, echo time (TE) = 2.86–2.98 msec, in-plane resolution of 1.25 × 1.25 mm, 1.25 mm slice thickness, inversion time = 900 msec and flip angle = 9°.

Voxel based morphometry analysis used SPM12 (www.fil. ion.ucl.ac.uk/spm). Images were first approximately aligned by coregistration to an average image in MNI space, before segmentation and calculation of total intracranial volume (TIV). After segmentation, a study-specific DARTEL template was created from the 8 patient scans and the 8 controls mostly closely matched in age on a patient by patient basis, using default parameters. All subject scans were then warped to this template. The templates were affine aligned to the SPM standard space using ‘Normalise to MNI space’ and the transformation applied to all individual grey-matter segments together with an 8 mm FWHM Gaussian smoothing kernel. The resulting images were entered into a full factorial general linear model with a single factor of group having two levels (patient or control), and age and TIV as covariates of no interest. This model was estimated in the classical manner, based on restricted maximum likelihood. Voxels were defined as atrophic if they were statistically significant at the cluster FWE *p* < .05 level, with an uncorrected cluster defining height of *p* < .001.

The same statistical model was then re-estimated using the Bayesian inference framework of SPM12 (Han & Park, 2018). This model was first assessed for areas of grey matter atrophy in SD, to ensure that the results of the classical frequentist approach could be replicated with Bayesian inference. Then, crucially, the model was inverted with the spm_bms_test_null function to look for brain areas where there was significant evidence against atrophy in SD. Thresholding was undertaken at Bayesian probability of the null >.7, with a minimum 1 cm^3^ cluster defining volume (Cope et al., 2017a).

To produce the supplementary material single subject atrophy maps for each patient, new full factorial general linear models were created, each containing the images from a single patient and all of the controls. Again the analysis contained age and TIV as covariates of no interest. This model was estimated in the classical manner, based on restricted maximum likelihood, and the resulting t-map exported for visualisation.

### 2.4 Magnetoencephalography data acquisition and preprocessing

MEG data were acquired with a 306-channel Vectorview system (Elekta Neuromag, Helsinki) with 102 magnetometers and 204 paired planar gradiometers. Data were digitally sampled at 1 kHz and high-pass filtered above .01 Hz. Throughout scanning, the 3D position of five evenly distributed head position indicator (HPI) coils was continuously monitored relative to the MEG sensors. The positions of these indicator coils, relative to overall head shape and the position of three anatomical fiducial points (nasion, left and right preauricular), were measured before scanning with a 3D digitiser (Fastrak Polhemus). Electrooculography data were also acquired to allow later data artefact removal.

MEG and HPI data were pre-processed in Neuromag Maxfilter 2.2 to perform Signal Source Separation (Taulu, Simola, & Kajola, 2005) for motion compensation and environmental noise suppression. All subsequent data analysis steps were undertaken in Matlab 2013a (The Mathworks Inc., 2015) using the software packages SPM12-r6906 (Wellcome Trust Centre for Neuroimaging, London, UK), FieldTrip (Donders Institute for Brain, Cognition, and Behaviour, Radboud University, Nijmegen, The Netherlands) and EEG lab (Swartz Center for Computational Neuroscience, University of California San Diego). Magnetometer and planar gradiometer data were subjected to separate independent component analyses for artefact rejection. Artefactual components were automatically identified by a conjunction of temporal correlation with electrooculography data and spatial correlation with separately acquired template data for blinks and eye movements.

The cleaned data were then sequentially epoched from −500 to 1500 msec relative to word onset; downsampled to 250 Hz; baseline corrected to the 100 msec before word onset; lowpass filtered below 40 Hz; merged across recording session; averaged using the SPM robust averaging algorithm, which produces an average after weighting individual epochs according to their consensus; and re-filtered below 40 Hz to remove high frequency components introduced by robust averaging. Planar gradiometer data pairs were root-mean-square combined; converted to scalp-time images; smoothed with a 10 mm spatial kernel and 25 msec temporal kernel; and finally masked for statistical analysis to time windows from −100 msec to 600 msec relative to the timing of standard word offset.

### 2.5 Sensor-space evoked analysis

The initial analysis of the contrast between standard and deviant words was undertaken in sensor space, for which the signal to noise ratio is higher than data in source space (Martín-Buro et al., 2016) and no a-priori specification of time windows of interest is required. To allow robust interpretation of laterality effects, this analysis was performed on the planar gradiometer data, for which signal magnitude at the scalp is maximal directly over the source of neural activity (Parkkonen, 2010, pp. 29–69). A flexible factorial design was specified in SPM12, allowing us to compensate for the difference in the number of individuals in control and patient groups, ensuring that unequal group sizes and differential variances did not produce any biases or false positives [for a discussion of this approach to unequal groups in neuroimaging see (McFarquhar, 2016)]. This design was estimated and interrogated across all participants for main effects of interest. The scalp location of peak statistical effect was identified on each side (left and right; in all cases p(FWE) was <.01). The time-courses of the sensor data extracted at each of these scalp locations was then compared across groups at every time point. This approach is superior to the extraction of time-courses from a single, gradiometer pair closest to the peak statistical effect, as it inherently controls for interindividual differences in head position relative to the detector array. Further, by virtue of spatial smoothing it includes weighted information from nearby sensors, reducing the effect of differential noise in any one superconducting quantum interference device (SQUID). In the results, scalp locations are given in the SPM coordinate system (Litvak et al., 2011), whereby the first dimension is left-right, with negative numbers being to the left of midline and positive numbers to the right of midline, and the second dimension is anteriorposterior, with positive numbers anterior of the scalp location overlying the anterior commissure and negative numbers posterior of it.

Crucially, this approach does not represent double dipping, as the location of interest for between-group comparison was defined by the orthogonal contrast of overall main effect, accounting for differences in group sizes and variances (Friston & Henson, 2006; Kilner, 2013; Kriegeskorte, Simmons, Bellgowan, & Baker, 2009).

When comparing extracted time-courses, a significant group × condition interaction was defined as at least seven consecutive time-points of *p* < .05, resulting in a sustained effect over≤28 msec, exceeding the temporal smoothing induced by lowpass filtering at 40 Hz.

Laterality effects in the analysis of deviant word endings were assessed through laterality quotients (Holland et al., 2012). These were calculated for every time-point outside of the baseline period for each individual separately as:

$$\text{laterality}\;\text{quotient}=\frac{S_{l}-S_{r}}{S_{l}+S_{r}}\times 100$$

where *S_l_* and *S_r_* are the magnitudes of the deviance effect at the same scalp locations as interrogated for the group by deviance interaction on each side. The laterality quotients were assessed at every time point both for difference from zero for each group separately, and for group by deviance interactions.

### 2.6 Source-space evoked analysis

Source reconstructions were undertaken (using SPM12) to localise the brain basis of any neurophysiological interaction between word ending and group that was statistically demonstrated in sensor space.

Single shell MEG forward models were created for each participant. First a brain mesh was created based on that subject's MRI scan. Individually recorded head shapes were then co-registered to this mesh using fiducial points and around 100 individually digitised scalp-surface points. Magnetometer and planar gradiometer data were combined (Henson, Mouchlianitis, & Friston, 2009) and group source inversion across all participants was undertaken with sLOR-ETA (Pascual-Marqui, 2002) across epochs of −100 msec–900 msec relative to spoken word onset. Within the time window of interest, condition estimates were computed in a 1–40 Hz frequency band and converted into images. These images were then subjected to statistical analysis, within a flexible factorial general linear model design identical to that employed for the sensor-space evoked analysis. This led to the creation of t-score maps contrasting the neural response to standard and deviant words, which were then thresholded for visualisation of the location of the effects already statistically demonstrated in sensor space.

## 3 Results

### 3.1 Voxel based morphometry

**Fig. 2:**
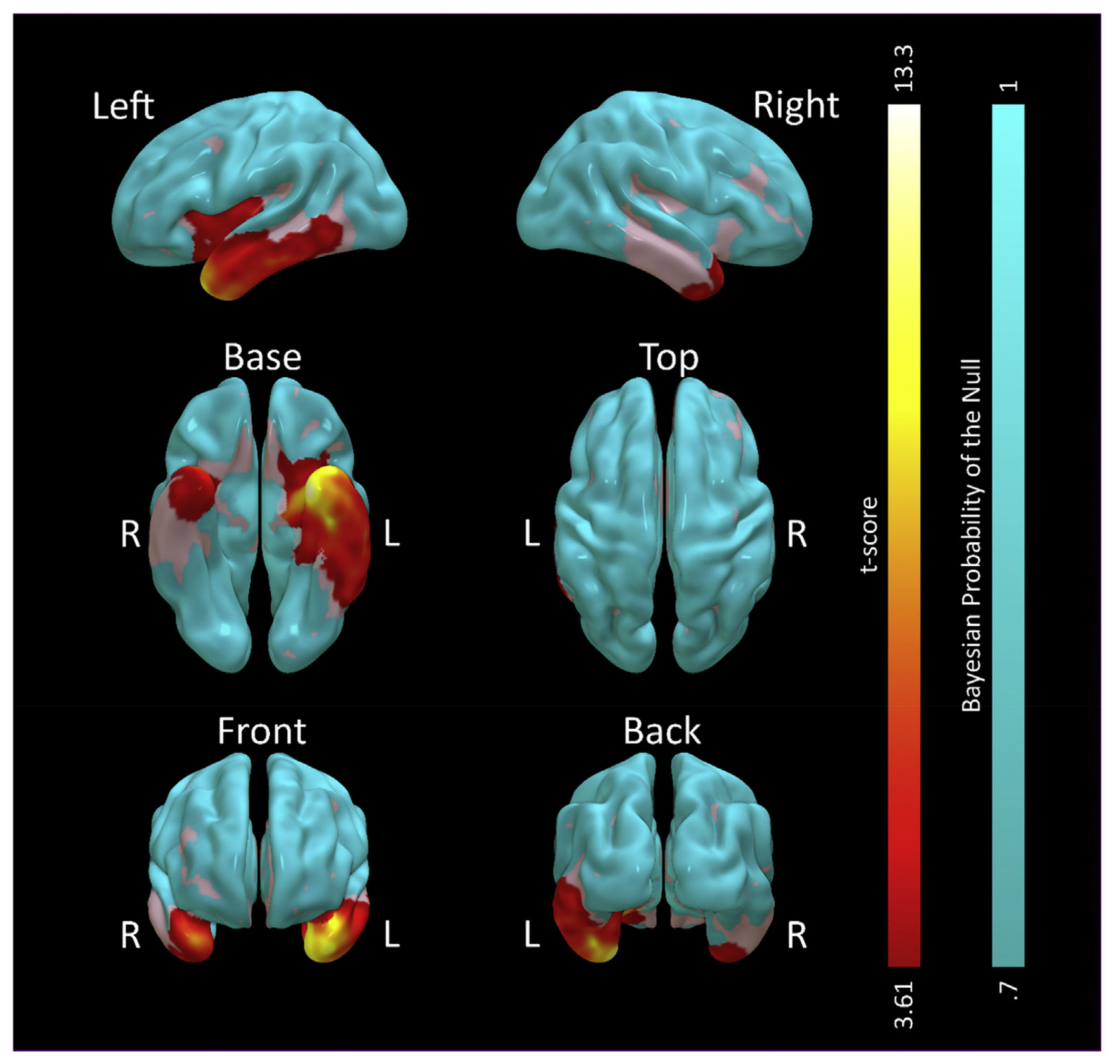
Voxel based morphometry statistical comparison of 8 participants with SD against 14 age-matched controls. Redyellow shaded areas represent t-scores for greater grey matter volume in the control group on the frequentist analysis, cluster thresholded at FWE p < .05 with a height threshold at uncorrected p < .001. No voxels demonstrated greater grey matter volume in the patient group. Cyan areas represent those that had strong evidence for normal grey matter volume in SD compared to controls on the Bayesian analysis (Bayesian probability of the null >.7, cluster volume>1 cm^3^). Uncoloured (grey) areas had no strong evidence for or against atrophy.

Using classical, frequentist, inference from statistical parametric mapping, voxel based morphometry (Fig. 2) demonstrated the expected pattern of SD, with predominant grey matter loss compared to the control group in the left ATL [peak (–29 1–40) t(18) = 13.34 FWE *p* < .001], with more posterior temporal regions affected to a lesser degree. Every patient displayed lower grey matter volume in left temporal pole than every control (patient range .261–.384 A.U., control range .446–.673 A.U). There was also atrophy of the same region on the right that was less marked in magnitude and extent [peak (36 14–32) t(18) = 8.05 FWE *p* = .004]. Right atrophy was present in all but one patient (patient range .263–.491 A.U., control range .470–.681 A.U). Volume loss of the left insula was also observed that exceeded the cluster defining height (as illustrated in Fig. 2) but was not significant at the corrected voxel level [peak (–33 148) t(18) = 5.12 FWE *p* = .29]. Grey matter volume elsewhere was not statistically different from control participants.

A Bayesian estimation of the same statistical model confirmed the results of the classical estimation, with grey matter loss in a similar distribution [log Bayes Factor (logBF) = 80.26, probability of no difference <.0001, in left ATLat (–29 1–40) and logBF = 31.12, probability of no difference <.0001, in right ATL at (36 14–32)]. Importantly, this analysis also demonstrated evidence for the null hypothesis in the frontal and parietal modules of the classical language network: logBF = −1.75, probability of no difference .85 in left frontal operculum at [−47 15 1]; logBF = −1.52, probability of no difference .83, in right frontal operculum at [47 19 −1]; logBF = −1.66, probability of no difference .84, in left supramarginal gyrus at [e55 –28 43]; and logBF = −.93, probability of no difference .72, in right supramarginal gyrus at [56 –28 46].

### 3.2 Overall magnetic response to standard words

**Fig. 3:**
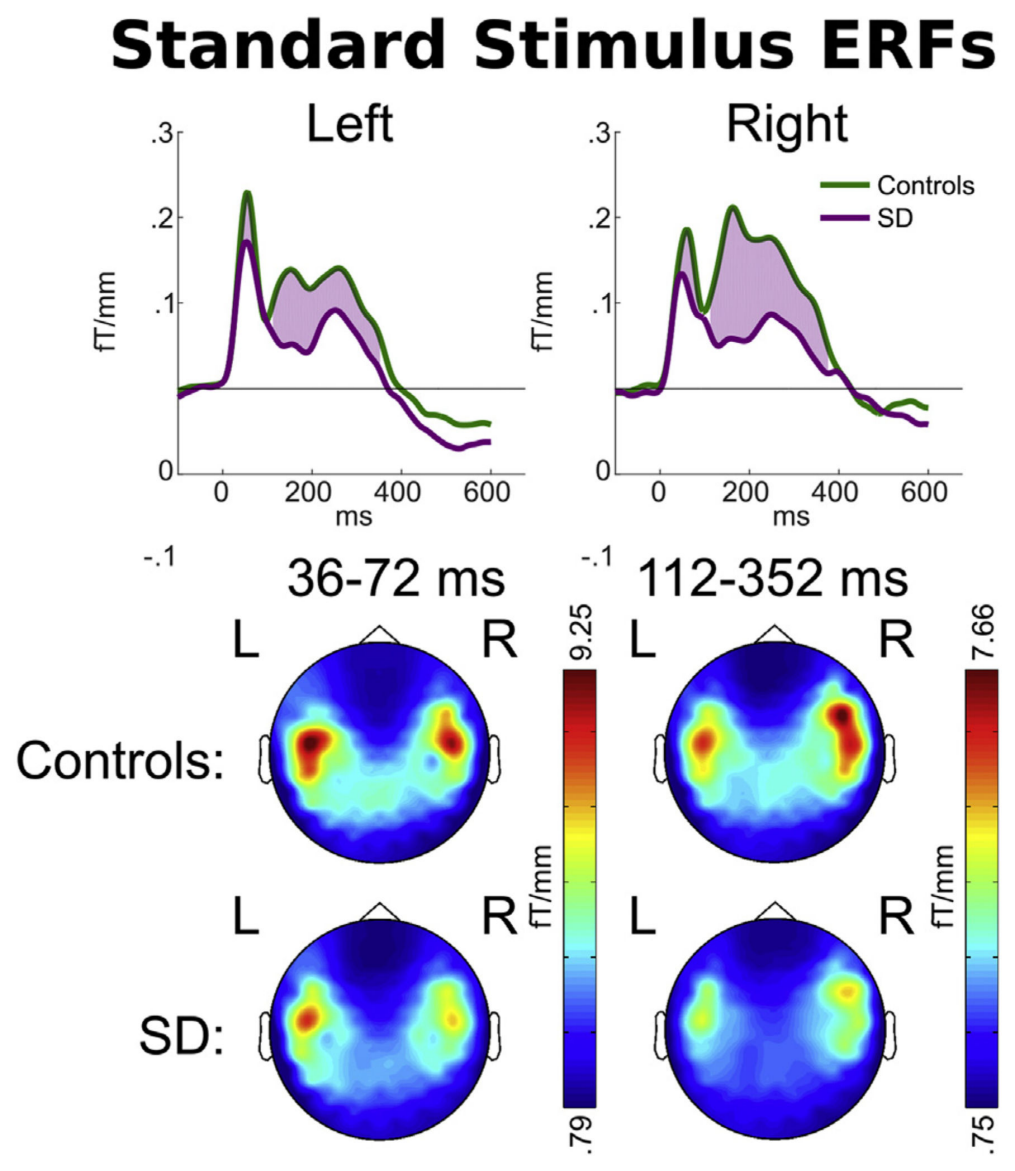
Upper: Magnetic field power recorded by planar gradiometers at the scalp locations of peak overall response [(−47, −9) and (47, −9)] to the standard word overlying each hemisphere. Responses are time-locked to word onset. Purple shading indicates time periods at which a statistical difference was observed in signal magnitude between patients and controls. Lower: Scalp signal topographies for each group, averaged within each period of statistically significant difference.

At the scalp locations of peak response overlying each hemisphere [(–42 –9) on the left, (42 –9) on the right, roughly overlying superior temporal lobe on each side], overall magnetic response to the three standard stimuli (2 words and 1 non-word) was significantly greater in the control group than the SD group in an early (36–72 msec) and a late (112–352 msec) time window relative to word onset (Fig. 3 upper). The distribution of this response was similar across the two groups (Fig. 3 lower).

### 3.3 Response to deviant word disambiguation

**Fig. 4:**
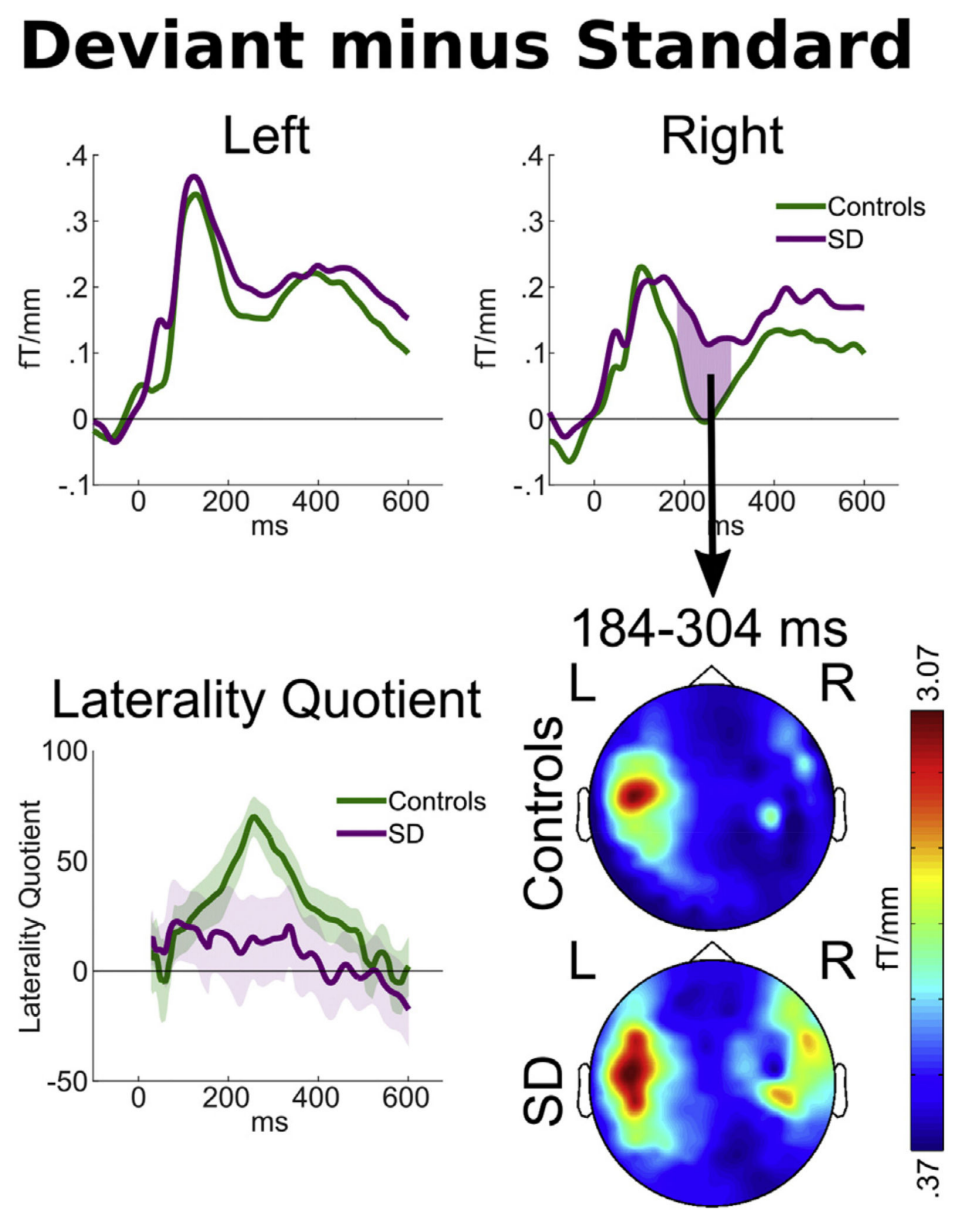
Upper: Magnetic field power recorded by planar gradiometers at the scalp locations of overall peak contrast between the average responses to all deviant words minus all standard words, relative to standard word offset. Pink areas indicate statistically significant group by deviance interactions as defined by *p* < .05 sustained for ≤7 samples, exceeding the duration of temporal smoothing. Lower left: The laterality quotient of the deviance response for each group. Calculated such that fully left sided deviant responses would be +100, fully right sided responses −100. The shaded areas around each line encompass ± one standard error. Lower right: Scalp signal topographies for each group, averaged across the period of the statistically significant group by condition interaction observed in the peak right-sided sensor.

Despite the group difference in overall magnetic power in response to standard words, both groups demonstrated peak responses to the overall contrast between standard and deviant word endings of similar magnitude, with a much larger response to deviant words at around 100–160 msec after stimulus disambiguation (Fig. 4 upper). As has been previously observed in younger participants (Holland et al., 2012), for the older controls the deviance response for word ending was significantly greater on the left than on the right during this early peak. Indeed, for controls the laterality quotient was significantly greater than zero (more activity on the left) for every time point from 128 to 440 msec [t(13) *p* < .05; peak t(13) = 7.71, *p* = 3.36 × 10^−6^ at 256 msec]. While patients demonstrated a deviance response of very similar average magnitude during this early time window (lines almost overlapping on both sides before 150 msec in Fig. 4 upper), due to the smaller group size and greater between-individual variability, the patient laterality quotient did not significantly differ from zero at any time point.

At later time windows (184–304 msec after standard word offset, which is 174–294 msec after the divergence point between/d/and/t/endings), a significant group by deviance interaction was observed in the right hemisphere, such that patients with SD demonstrated a larger difference between deviant and standard stimuli in the right hemisphere [peak t(21) = 3.13, *p* = .0050]. Scalp topographies of average power during this period (Fig. 4 lower) confirmed that this was not an effect restricted to the peak location, but rather represented a more general shift from highly left lateralised responses in controls to bilateral processing in patients with SD. Indeed, patients and controls demonstrated significantly different laterality quotients between 232 and 292 msec after standard word offset [peak t(21) = 2.90, *p* = .0086 at 256 msec].

**Fig. 5:**
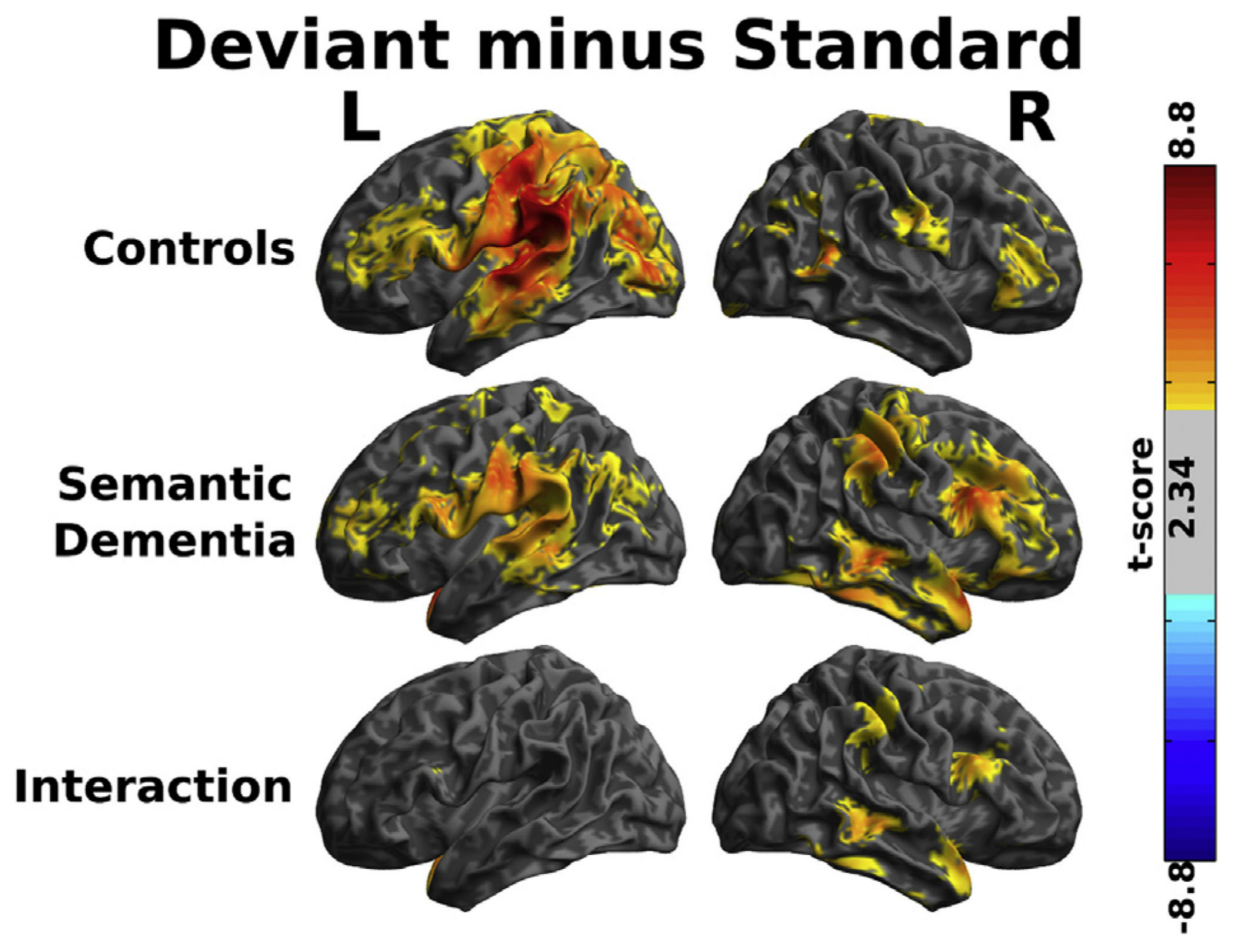
Source reconstructions of the contrast between standard and deviant words between 240 and 280 msec after the offset of the standard word, the time window during which the largest group by deviance interaction was demonstrated in sensor space (cf Fig. 4). Shaded areas represent t-scores thresholded for visualisation at t > 2.34 (equivalent to uncorrected *p* < .01). Two-tailed statistical tests were performed, but all surviving contrasts were greater in the deviant than the standard, and (for the third panel) the effect of deviance was greater in the patients than the controls.

We performed source localisations to assess the brain basis of the group difference in deviance response that we have statistically demonstrated in sensor space. Consistent with the scalp topographies in Fig. 4, between 240 and 280 msec after standard word offset healthy controls demonstrated a highly lateralised response predominantly involving left planum temporale and parietal lobe, with some involvement of inferior frontal regions (Fig. 5 upper). Patients with SD demonstrated similar left sided responses, which were of lower average magnitude than controls, but not to a statistically significant degree. However, they had much more extensive activation of the right hemisphere (Fig. 5 middle), again consistent with the sensor-space results presented in Fig. 4. The voxelwise group by condition contrast (Fig. 5 lower) demonstrated above-threshold clusters, with peak differences assessed by the Neuromorphometrics atlas to be in right temporal pole [(48 14 –2] t(357) = 4.62], right middle temporal gyrus [(48 –32 –6) t(357) = 4.15], right frontal operculum [(54 16 26) t(357) = 4.04], right inferior temporal gyrus [(54 –44 –26) t(357) = 3.50], and right supramarginal gyrus [(56 –28 46) t(357) = 3.21], in what might be deemed right sided analogues of a classical map of the brain regions involved in language (Friederici, Chomsky, Berwick, Moro, & Bolhuis, 2017). In all cases where these right-hemispheric differences were observed, patients with SD demonstrated equal or greater modulation of brain activity as a function of word ending than controls, despite the lower overall power of their magnetoencephalography response to spoken words (Fig. 3).

### 3.4 Response differences according to standard word identity

Our paradigm was designed to assess the brain response to the disambiguation of deviant words, and as such includes a high degree of predictability and repetition in the presentation of standard words. However, there is some evidence that even highly repetitive standard word presentation provokes the automatic activations of word-specific memory traces that are unaffected by attention or active task. We therefore present in supplementary materials, and with appropriate caveats, our analyses and interpretations of the standard word MEG data from SD patients relative to healthy age-matched controls.

## 4 Discussion

There are three principal results of this study. First, severe degeneration of the anterior temporal lobes leads to wide-spread abnormal engagement of right-hemisphere analogues of the language network, during processing of word identity (between 174 –294 msec after the divergence point at which stimuli were disambiguated). There was no change in the laterality or magnitude of the peak early response to deviant word endings, occurring approximately 115 msec after stimulus disambiguation. This is consistent with a framework in which auditory information passes from primary auditory areas (intact in SD) to ATL so as to engage the left-lateralised processing of word identity. Second, we identified diaschisis – that is, degeneration of the neural architecture in anterior temporal lobes alters activity in extra-temporal brain regions that were not themselves significantly atrophic, either as a direct result of changes in reciprocal connectivity or as a compensatory phenomenon. Third, we found that in healthy elderly adults, the processing of deviant word endings that change word identity and meaning is strongly left lateralised, as in young healthy adults (Holland et al., 2012).

Our results definitively answer the question posed in the Introduction, demonstrating a strongly left-lateralised pattern of activity in healthy controls that shifted to a bilateral pattern in the SD patients. Note that this is not a necessary outcome: of course the brain response in patients will be lower or even largely absent in the lesioned region, but the further consequence of this might be either no increased activity anywhere, or higher responses in other, less-damaged, left-sided regions. Of particular relevance to the current study is the fMRI finding by Maguire, Kumaran, Hassabis, and Kopelman (2010) that the usual left-dominant brain activity underlying retrieval of autobiographical memories in controls changed in SD to a pattern of bilateral activity.

A similar question regarding the laterality of brain bases for language processing, whether it represents a compensatory phenomenon, and whether such compensation is effective, is often asked (but rarely answered in a definitive manner) in relation to post-stroke aphasia resulting from lesions in classic left-sided language regions. Specifically, is it mainly right-hemisphere activity or is it activity in left-hemisphere areas not specialised for language that mediates recovery? The most likely answer is probably that both of these phenomena occur depending on the nature and extent of the lesion (Karbe et al., 1998; Price et al., 1998). Unsurprisingly, activity in these additional atypical areas does not properly compensate for the reduced response in typical regions: the patients' performance is always still impaired. Although we did not test the SD patients in the current study on their knowledge of the stimulus words, we know from substantial previous research and clinical experience in SD that the patients would easily repeat PLAY or PLAYED or PLATE, but would not necessarily know the words' identities in the full sense of understanding their meanings. There is evidence from non-human primates that right sided frontoetemporal interactions support structured sequence learning (Wilson et al., 2013; Wilson, Marslen-Wilson, & Petkov, 2017), and that similar analysis strategies are employed to learn artificial grammars in healthy (Wilson, Smith, & Petkov, 2015) and aphasic (Cope et al., 2017b) human listeners. However, these paradigms were explicitly designed to be independent of semantics, and hence represent a very different cognitive task to that described here. While it seems likely that the patients' additional right-hemisphere activations contribute to the process of acoustic analysis, helping to preserve word repetition ability, they do not necessarily enable word comprehension.

In supplementary materials we present some analyses of the responses to standard words that suggest that, in SD, the brain processing of real words and word-like non-words becomes increasingly similar. As mentioned in the Introduction, SD patients are impaired at distinguishing between specially designed words and non-words in visual lexical decision (Patterson et al., 2006; Rogers et al., 2004). When a real word like FRUIT with rather atypical spelling was paired with a more typically spelled non-word homophone (FRUTE) and the patients were asked to choose the real word, all 22 SD patients had abnormal accuracy, and the more advanced cases tended to prefer the typical non-word to the atypical word as ‘the real thing’. Patterson et al. (1994) and Knott et al. (1997) studied immediate serial recall of short word sequences by SD patients, under three conditions: real words that each patient still ‘knew’ or understood; real words that he or she no longer understood; and word-like non-words. Successful recall of the real-but-‘unknown’ words was at a level intermediate between real-“known” words and non-words. Finally, in tasks of reading aloud briefly presented written words and tasks of identifying words from oral spelling (e.g., “what does C,H,U,R,C,H spell?”), both SD patients and stroke patients with posterior left-hemisphere lesions resulting in pure alexia made many errors (Cumming, Patterson, Verfaellie, & Graham, 2006). Strikingly, however, virtually all of the error responses by the pure alexic patients in both tasks were other similar real words, whereas the majority of the errors by the SD patients were orthographically and phonological similar non-words. All three of these studies were purely behavioural experiments, demonstrating significantly reduced ability to distinguish between real, meaningful words and plausible non-words. The current study represents an important advance by demonstrating a brain-basis for this phenomenon, with a loss of the normal laterality of spoken word processing.

There are a number of limitations to our study. The presentation of stimuli was passive. This was a design choice, made to reduce the difficulties that arise when patients with semantic impairment are required to comprehend and retain task instructions. However, it naturally restricts our ability to assess the direct cognitive consequences of the abnormal neuronal activity we observe. Secondly, the sample size was relatively small. SD is a very rare illness (Coyle-Gilchrist et al., 2016) and, to maximise interpretability, an effort was made to recruit individuals with early stage disease and atrophy restricted to anterior temporal lobes. While we were adequately powered to detect the very large effect sizes that we have demonstrated in relation to severe temporal polar atrophy, larger study numbers may provide greater support for the generalisation of inferences to the broader SD population. Thirdly, while we conclude that our observations of neuronal diaschisis (right sided extra-temporal brain activity) are due to ATL atrophy, we are unable to be definitive as to whether it is specifically left ATL that is necessary, or the degree of contribution from the mild right ATL atrophy. While every patient with SD had lower left anterior temporal lobe grey matter volume than every control (i.e., the group ranges were non-overlapping), most also had mild right anterior temporal lobe atrophy.

## 5 Conclusions

Our results indicate that ATL performs a necessary role in the left-lateralisation of linguistic processing of words, which represents an efficiency saving compared to the bilateral processing of non-words. We measured abnormal activity in extra-temporal brain regions that we have demonstrated, through Bayesian voxel based morphometry, are not atrophic. It therefore seems likely that, although SD patients have no measurable damage in these caudal and dorsal regions, their significant atrophy in the rostral and ventral temporal lobes would alter both forward and backward activations between the two sets of regions, resulting in diaschisis. We suggest that this abnormal, perhaps compensatory, reliance on the right hemisphere as a consequence of ATL atrophy results in automatic word identity processing becoming predominantly acoustic/phonetic rather than lexical.

## Supplementary Material

Supplementary data to this article can be found online at https://doi.org/10.1016/j.cortex.2019.12.025.

All supplementary files included with this article

## Figures and Tables

**Table 1:** Participant demographics. Mean (standard deviation). ACE-R = Addenbrooke's cognitive examination, revised edition. MMSE = Mini-Mental State Examination.

Group	Number	Age	Gender	ACE-R	MMSE
SD	9	68 (6)	3F 6M	57 (12)	24 (3)
Control	14	67 (7)	11F 3M	−	−
