# Supplementary material for: Anterior temporal lobe is necessary for efficient lateralised processing of spoken word identity
Source: Cortex. Author manuscript; Available in PMC 2020 Nov 1. (PMC7253293; doi:10.1016/j.cortex.2019.12.025)
Supplement: All supplementary files included with this article [file EMS86348-supplement-All_supplementary_files_included_with_this_article_.zip › 1-s2.0-S0010945220300162-mmc1.docx]

**Supplementary Analysis of Standard Words**

Hypotheses

The high degree of predictability and repetition in the experiment meant that it was not designed to assess for differential processing of standard words. However, there is some evidence that even highly repetitive standard word presentation provokes the automatic activations of word-specific memory traces that are unaffected by attention or active task. Repetition is known to lead to response suppression, but this would lead to a reduction in our ability to detect effects rather than creating effects that do not exist. Therefore, to some extent our design helps to highlight the most robust, automatic stages of speech parsing. It has previously been demonstrated that similar patterns of lexical responses are acquired with this technique and in similar protocols that lack repetition (MacGregor *et al*, 2012).

We therefore present here, in supplementary materials, and with appropriate caveats, our analyses of the standard word MEG data from SD patients relative to healthy age-matched controls addressed to address the questions of whether degeneration of the left ATL would result in:

1) increased similarity in the distribution of the brain response to words and word-like non-words?

2) an overall shift in brain activity from areas implicated in word processing to those involved in the analysis of non-linguistic acoustic features?

Methods

MEG pre-processing was identical to that employed for the analysis of standard word offset, other than that masking before statistical was from -100 to 900ms relative to standard word onset, allowing for the analysis of non-overlapping epochs (the inter-stimulus interval was 1 second).

In contrast to the analysis of deviant word endings, where a two-stage inference was undertaken based on sensor space statistics reconstructed in source space, here primary statistical analysis was performed in source space. This was possible because there were four times as many repetitions of each stimulus, which allowed adequate signal-to-noise ratio, and a there was a precisely-defined temporal window of interest based on overall contrast magnitude (supplementary figure 1).

Results

We explored the consequences of left ATL neurodegeneration for the neuronal processing of standard words. An initial assessment of magnetic response power across all gradiometers revealed that the only large differences between word pairs occurred in early time windows, between 50 and 70ms after word onset (*Supplementary figure 1*), corresponding to the early peak in overall brain response (*Figure 3* *upper*).


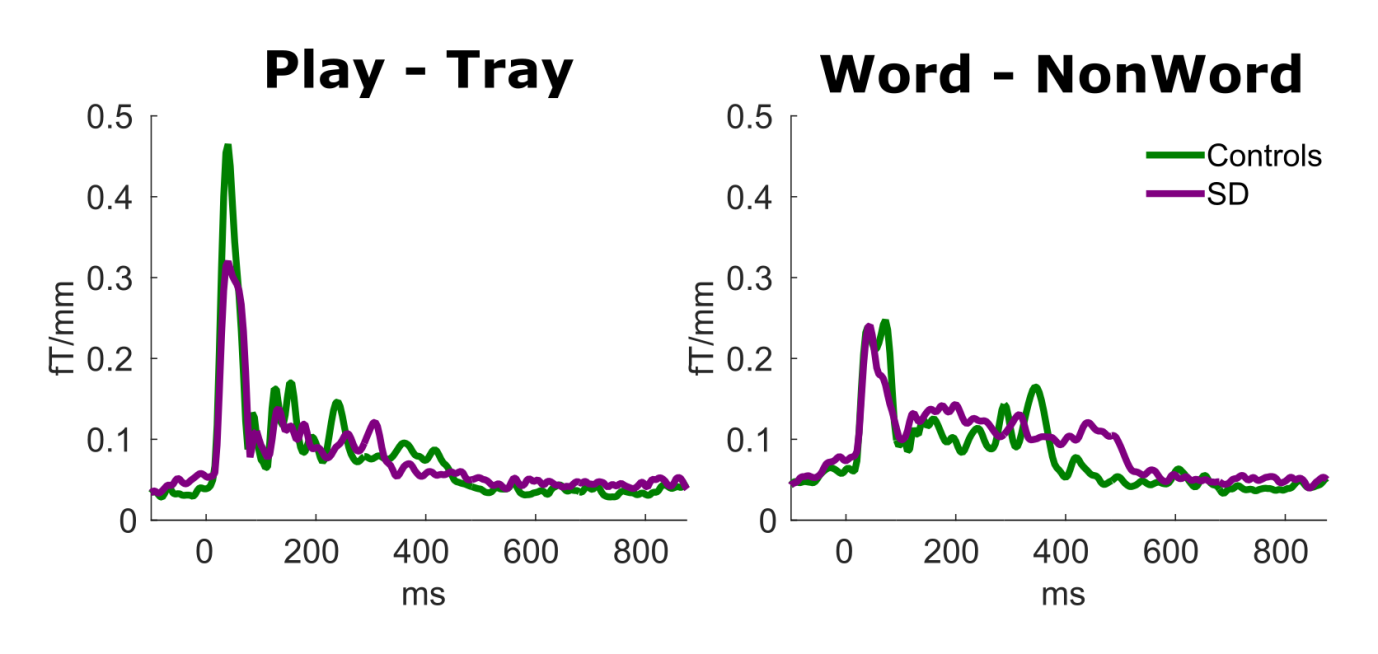


*Supplementary Figure 1: Overall root-mean-squared (i.e. unsigned in every location) difference in magnetic power detected by planar gradiometers across the whole scalp as a function of word identity, relative to the onset of the standard word.*

To test for the presence of interactions between group and standard word identity we therefore performed source reconstructions in the 50-70ms time window and interrogated these with a primary whole-brain SPM.

For the contrast between real words (PLAY + TRAY) and the non-word KWAY (*Supplementary figure 2*), controls displayed significantly greater brain activity for non-words than words bilaterally (*Supplementary table 1*). The only significant interaction between this contrast and group was in right hemisphere regions surrounding primary auditory cortex and planum temporale in superior temporal lobe. Controls demonstrated a significantly greater difference in activity between words and non-words in these regions than patients with SD. Indeed, although the time window of reconstruction was chosen to capture the peak neuronal response across the whole brain, no individual voxels survived statistical thresholding in the SD group. In this group the non-significant locations of peak contrast in each hemisphere were: left postcentral gyrus at MNI [-60, -6, 22], t(357)=3.56, p(FWE)=0.43, and right frontal operculum at MNI [46, 14, 8], t(357)=3.92, p(FWE)=0.16.


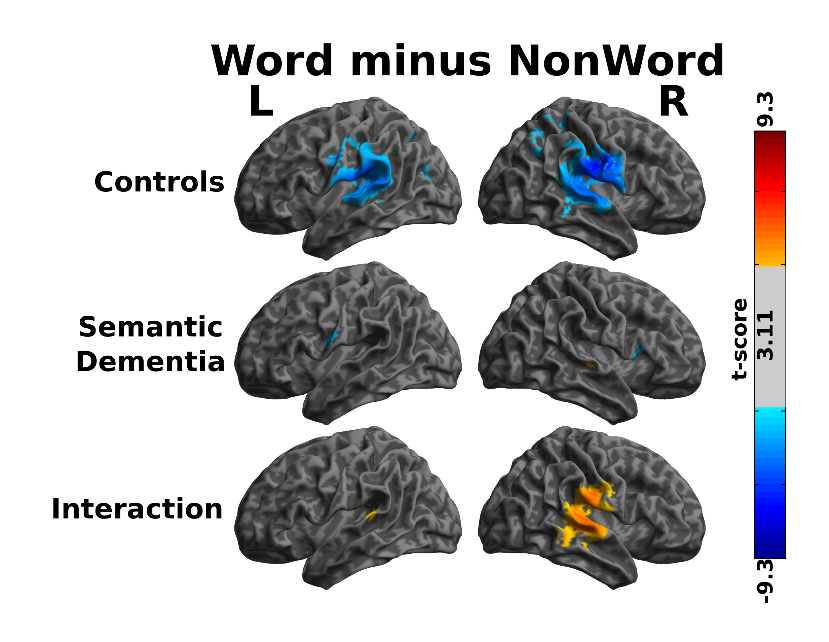


*Supplementary figure 2: Source reconstructions of the contrast between standard real words and non-words 50-70ms after onset, the time window during which the largest effect of word identity was demonstrated across the whole brain (cf Supplementary figure 1). Shaded areas represent t-scores thresholded at uncorrected p<0.001 (t>3.11). In the lower right panel red shaded regions represent greater contrast in controls; no voxels demonstrated greater contrast in SD.*

For the contrast between the verb PLAY and the noun TRAY*,* participant group by word identity interactions were demonstrated in both directions (*Supplementary figure 3*, *Supplementary table 1*). All interactions were in the left hemisphere. Patients with SD displayed a greater effect of word identity in regions surrounding primary auditory cortex and planum temporale in posterior superior temporal lobe. Controls, on the other hand, demonstrated a greater effect of word identity around supramarginal gyrus in parietal lobe.

Overall, therefore, both groups were characterised by a left-lateralised effect of real word identity, but group by word interactions revealed that in SD this was greater around primary auditory regions in temporal lobe while in controls it was greater around parietal regions that some researchers have proposed to play a significant role in linking phonological analysis to meaning (Robson *et al*, 2013, Robson *et al*, 2017). Further, relative to the SD group, controls displayed a significantly greater response to non-words than to real words across superior temporal lobe and planum temporale, especially on the right. Indeed, source reconstructions demonstrated no significant clusters of activity in the SD group that differed between real words and non-words.

*
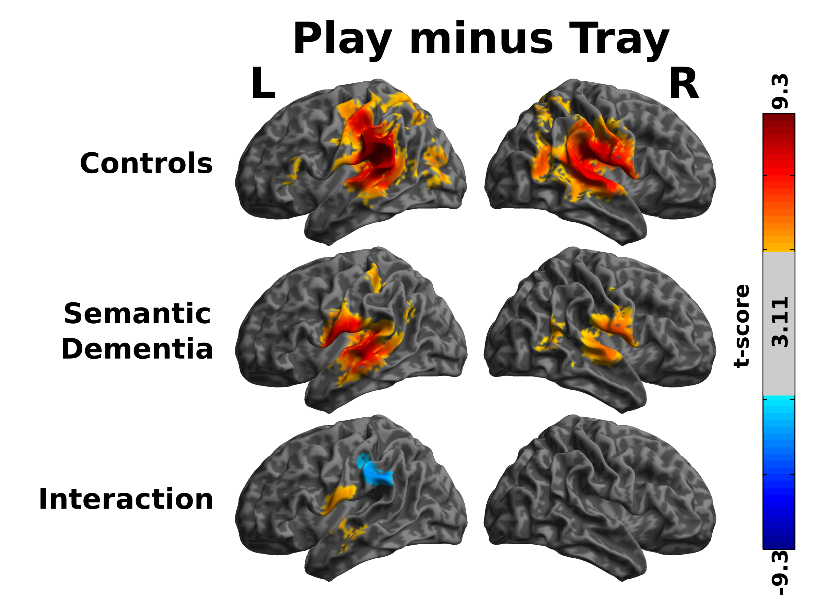
*

*Supplementary figure 3: Source reconstructions of the contrast between the two standard real words 50-70ms after onset, the time window during which the largest effect of word identity was demonstrated across the whole brain (cf Supplementary figure 1). Shaded areas represent t-scores thresholded at uncorrected p<0.001 (t>3.11).In the row representing the group by condition interaction control responses were subtracted from SD responses such that blue shaded areas represent greater contrast in the controls and red shaded areas represent greater contrast in SD.*

|  | Word minus Non-Word | | | Play minus Tray | | |
| --- | --- | --- | --- | --- | --- | --- |
|  | **MNI** | **t-score** | **P(FWE)** | **MNI** | **t-score** | **P(FWE)** |
| Controls | 54 -14 24 | 7.31 | <0.001 | -62 -28 26 | 9.28 | <0.001 |
|  | -52 -30 18 | 5.82 | <0.001 | 60 -18 22 | 8.50 | <0.001 |
|  | | | | | | |
| SD | No significant voxels | | | -46 -18 4 | 8.27 | <0.001 |
|  |  |  |  | 64 -10 14 | 5.53 | <0.001 |
|  | | | | | | |
| Interaction: |  |  |  |  |  |  |
| Controls greater | 64 -20 2 | 5.16 | 0.001 | -56 -40 32 | 4.42 | 0.030 |
|  | No significant left sided voxels | | | No significant right sided voxels | | |
|  | | | | | | |
| SD greater | No significant voxels | | | -46 -18 0 | 5.24 | 0.001 |
|  |  |  |  | No significant right sided voxels | | |

*Supplementary table 1: Voxels of peak statistical significance in each hemisphere from the whole-brain corrected SPM contrasts illustrated in Supplementary figures 2 and 3.*

Discussion

Our first supplementary question was whether we would observe differences in healthy listeners’ brain activity between real, familiar, meaningful words compared to word-like non-words, and whether such differences would be attenuated or absent in SD. Indeed this was the case: our statistical analysis of source-space data (see supplementary figure 2 and supplementary table 1) confirm that where the controls showed significant activation differences in response to words compared to non-words, the SD patients had no such differences, and there was an interaction between lexical status and diagnostic group. This is in keeping with previous observations suggesting that, in SD, the brain processing of real words and word-like non-words becomes increasingly similar. As mentioned in the Introduction, SD patients are impaired at distinguishing between specially designed words and non-words in visual lexical decision (Rogers *et al*, 2004, Patterson *et al*, 2006). When a real word like FRUIT with rather atypical spelling was paired with a more typically spelled non-word homophone (FRUTE) and the patients were asked to choose the real word, all 22 SD patients had abnormal accuracy, and the more advanced cases tended to prefer the typical non-word to the atypical word as ‘the real thing’. Patterson *et al* (1994) and Knott *et al* (1997) studied immediate serial recall of short word sequences by SD patients, under three conditions: real words that each patient still ‘knew’ or understood; real words that he or she no longer understood; and word-like non-words. Successful recall of the real-but-‘unknown’ words was at a level intermediate between real-“known” words and non-words. Finally, in tasks of reading aloud briefly presented written words and tasks of identifying words from oral spelling (e.g., “what does C,H,U,R,C,H spell?”), both SD patients and stroke patients with posterior left-hemisphere lesions resulting in pure alexia made many errors (Cumming *et al*, 2006). Strikingly, however, virtually all of the error responses by the pure alexic patients in both tasks were other similar real words, whereas the majority of the errors by the SD patients were orthographically and phonological similar non-words. All three of these studies were purely behavioural experiments, demonstrating significantly reduced ability to distinguish between real, meaningful words and plausible non-words. The current study represents an important advance by demonstrating a brain-basis for this phenomenon.

Finally, we asked whether there would be an overall shift in brain activity for the SD patients from areas implicated in normal word processing to those involved in acoustic feature analysis. This too was supported by the findings: group by word interactions demonstrated that the effect of word identity in SD was greater around auditory regions in superior temporal lobe while in controls it was greater around parietal regions (see Figure 8 and Table 2). Although the role of left parietal areas in word processing is not yet well understood or agreed, some authors have suggested that they support the link between auditory processing of words and their meanings (Robson *et al*, 2013, Robson *et al*, 2017), the combination of semantic concepts (Price *et al*, 2015) and the integration of lexical and semantic information (Price *et al*, 2016). Left posterior superior temporal cortex is particularly implicated in phonemic processing. It might be that less efficient phonemic analysis in the patient group, as indicated by increased activity in these brain regions, results in a failure to route lexical information to the more dorsal parietal region, which displayed greater activity in the control group.

It is important to caveat that the observed differences in brain activity for standard words were in an early time window from 50-70ms after stimulus onset, during which there was a difference in acoustic power between the stimuli (figure 1). However, this difference was identical between groups, and our analysis strategy here relies on the interaction between word identity and group. These early time windows might classically be thought to be pre-semantic, however our paradigm here was highly repetitious and the identity of the standard word highly predictable. For these reasons we present these results and discussion in the supplementary materials, as potentially interesting observations meriting further investigation in more natural listening environments, without losing focus on the primary aim of the study, which was to investigate the brain response to the disambiguation of word identity.

References:

Cumming TB, Patterson K, Verfaellie M, Graham KS. One bird with two stones: Abnormal word length effects in pure alexia and semantic dementia. Cogn Neuropsychol. 2006;23(8):1130-61.

Knott R, Patterson K, Hodges JR. Lexical and semantic binding effects in short-term memory: Evidence from semantic dementia. Cogn Neuropsychol. 1997;14(8):1165-216.

MacGregor LJ, Pulvermüller F, Van Casteren M, Shtyrov Y. Ultra-rapid access to words in the brain. Nature Communications. 2012;3:711.

Patterson K, Graham N, Hodges JR. The impact of semantic memory loss on phonological representations. J Cogn Neurosci. 1994;6(1):57-69.

Patterson K, Ralph MAL, Jefferies E, Woollams A, Jones R, Hodges JR, et al. “Presemantic” cognition in semantic dementia: Six deficits in search of an explanation. J Cogn Neurosci. 2006;18(2):169-83.

Price AR, Bonner MF, Peelle JE, Grossman M. Converging evidence for the neuroanatomic basis of combinatorial semantics in the angular gyrus. J Neurosci. 2015;35(7):3276-84.

Price AR, Peelle JE, Bonner MF, Grossman M, Hamilton RH. Causal evidence for a mechanism of semantic integration in the angular gyrus as revealed by high-definition transcranial direct current stimulation. J Neurosci. 2016;36(13):3829-38.

Robson H, Grube M, Ralph MAL, Griffiths TD, Sage K. Fundamental deficits of auditory perception in Wernicke's aphasia. Cortex. 2013 Jul-Aug;49(7):1808-22.

Robson H, Pilkington E, Evans L, DeLuca V, Keidel JL. Phonological and semantic processing during comprehension in Wernicke's aphasia: An N400 and Phonological Mapping Negativity Study. Neuropsychologia. 2017;100:144-54.

Rogers TT, Lambon Ralph MA, Hodges JR, Patterson K. Natural selection: The impact of semantic impairment on lexical and object decision. Cogn Neuropsychol. 2004;21(2-4):331-52.
